# Editorial: Finding New Epigenomics and Epigenetics Biomarkers for Complex Diseases and Significant Developmental Events With Machine Learning Methods

**DOI:** 10.3389/fgene.2022.850367

**Published:** 2022-01-27

**Authors:** Lu Xie, Tao Huang, Michael Liebman, Yu-Hang Zhang

**Affiliations:** ^1^ Shanghai Center for Bioinformation Technology, Shanghai, China; ^2^ Bio-Med Big Data Center, CAS Key Laboratory of Computational Biology, Shanghai Institute of Nutrition and Health, Chinese Academy of Sciences, Shanghai, China; ^3^ IPQ Analytics, Philadelphia, PA, United States; ^4^ Channing Division of Network Medicine, Brigham and Women’s Hospital, Boston, MA, United States

**Keywords:** machine learning, epigenomics and epigenetics, biomarkers, complex diseases, developmental events

**Editorial on the Research Topic**

**Finding New Epigenomics and Epigenetics Biomarkers for Complex Diseases and Significant Developmental Events with Machine Learning Methods**

The epigenome has been regarded as one of the most important regulators for genome on its functioning and downstream regulation, therefore, the maintenance of stable and normal functioning epigenome is quite crucial for living organisms. However, the epigenome changes over time, either naturally or triggered by various endogenous and exogenous factors like environment, disease state and infections. The alteration of the epigenome may be crucial intermediate between environments and biological phenotypes during development and pathogenesis. Therefore, developmental or pathological alterations can also be monitored using epigenome characteristics, which is exactly what epigenetics and epigenomics studies focus on.

Currently, studies on epigenetics and epigenomics mainly focused on the plasticity of epigenome during two major biological processes: development and pathogenesis, both of which are inextricably linked to the environment through the epigenetic modifications. From the research layers, current epigenomic and epigenetic studies can be further divided into multiple layers: 1) direct methylation on DNA molecules; 2) histone protein modification; 3) chromatin structure and 4) related noncoding RNAs. Integration all layers of epigenomics and epigenetics studies, the ultimate research goal in this field is to reveal the specific role of epigenome during the development and pathogenesis of human beings and explain the related biological mechanisms using typical epigenetics/epigenomics biomarkers.

Biologically, epigenetics and epigenomics describe complex interactions between environment and genomics, resulting in diverse modifications on histone and DNA molecules. With the development of detecting techniques (like microarray and Methyl-Seq), an explosive increase occurs in epigenetics and epigenomics data. To handle such massive complex data, machine learning models have been introduced in the analyses on data at this omics-level and contribute to the identification of potential disease/developmental events associated epigenetic biomarkers. However, several restrictions and challenges still remain in current epigenetics and epigenomics studies:

(1) For most epigenomics studies, patients are hard to recruit (comparing to normal controls), lacking samples with diseases characteristics;
(2) For each epigenome, epigenomic alterations with biological significance is highly imbalanced distributed across the genome, making it hard for us to detect;
(3) Comparing to the sample number, methylation sites targeted by current probes are too many, forming a matrix with much more variables than samples. Larger datasets and pre-modeling features selection may be potential solutions for current restrictions on epigenetics and epigenomics studies.

In this research topic, we focused on the application of machine learning models on data at epigenomics and epigenetic levels to identify potential biomarkers for complex diseases/developmental events, including cancers. There are 33 studies in this special issue.

We hope this collection will inspire the epigenomics and epigenetics researchers to use machine learning methods for biomarker identification and regulatory mechanism investigation.

